# Design of Decorated Self-Assembling Peptide Hydrogels as Architecture for Mesenchymal Stem Cells

**DOI:** 10.3390/ma9090727

**Published:** 2016-08-26

**Authors:** Annj Zamuner, Marta Cavo, Silvia Scaglione, Grazia Maria Lucia Messina, Teresa Russo, Antonio Gloria, Giovanni Marletta, Monica Dettin

**Affiliations:** 1 Department of Industrial Engineering, University of Padua, Padua 35131, Italy; annj.zamuner@phd.unipd.it; 2 National Research Council (CNR)—Institute of Electronics, Computer and Telecommunication Engineering, Genoa 16149, Italy; cavomarta@gmail.com (C.M.); silvia.scaglione@ieiit.cnr.it (S.S.); 3 Department of Informatics, Bioengineering, Robotics and System Engineering (DIBRIS), University of Genoa, Genoa 16145, Italy; 4 Laboratory for Molecular Surfaces and Nanotechnology (LAMSUN), Department of Chemical Sciences, University of Catania and CSGI, Catania 95125, Italy; grmessi@unict.it (G.M.L.M.); gmarletta@unict.it (G.M.); 5 Institute of Polymers, Composites and Biomaterials—CNR, Naples 80125, Italy; teresa.russo@unina.it (T.R.); angloria@unina.it (A.G.)

**Keywords:** self-assembling peptides, mesenchymal stem cells, bio-transamination, chemoselective ligation, IGF-1, vitronectin, RGD

## Abstract

Hydrogels from self-assembling ionic complementary peptides have been receiving a lot of interest from the scientific community as mimetic of the extracellular matrix that can offer three-dimensional supports for cell growth or can become vehicles for the delivery of stem cells, drugs or bioactive proteins. In order to develop a 3D “architecture” for mesenchymal stem cells, we propose the introduction in the hydrogel of conjugates obtained by chemoselective ligation between a ionic-complementary self-assembling peptide (called EAK) and three different bioactive molecules: an adhesive sequence with 4 Glycine-Arginine-Glycine-Aspartic Acid-Serine-Proline (GRGDSP) motifs per chain, an adhesive peptide mapped on h-Vitronectin and the growth factor Insulin-like Growth Factor-1 (IGF-1). The mesenchymal stem cell adhesion assays showed a significant increase in adhesion and proliferation for the hydrogels decorated with each of the synthesized conjugates; moreover, such functionalized 3D hydrogels support cell spreading and elongation, validating the use of this class of self-assembly peptides-based material as very promising 3D model scaffolds for cell cultures, at variance of the less realistic 2D ones. Furthermore, small amplitude oscillatory shear tests showed that the presence of IGF-1-conjugate did not alter significantly the viscoelastic properties of the hydrogels even though differences were observed in the nanoscale structure of the scaffolds obtained by changing their composition, ranging from long, well-defined fibers for conjugates with adhesion sequences to the compact and dense film for the IGF-1-conjugate.

## 1. Introduction

**Figure 1:**
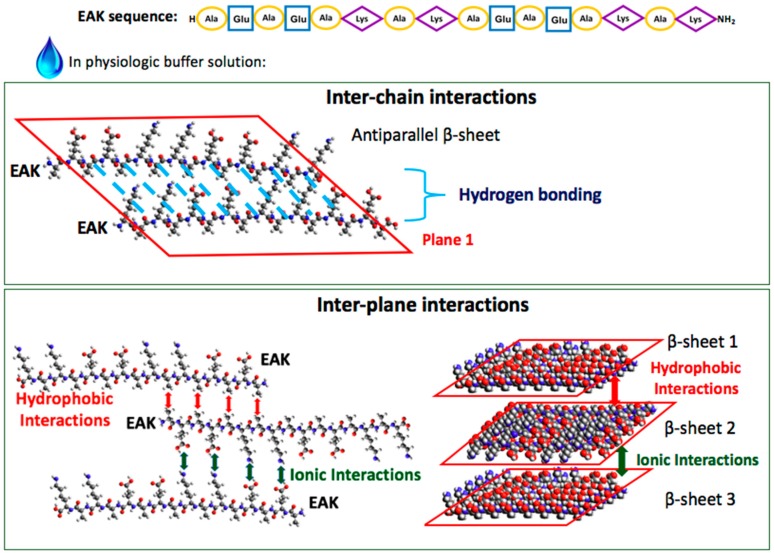
Scheme of peptide self-assembling. The sequence of the self-assembling peptide used in this study (called EAK): hydrophobic amino acids are circled in yellow, hydrophilic amino acids are in blue squares (acid amino acids, negatively charged at neutral pH) or in violet diamonds (basic amino acids, positively charged at neutral pH).

The design of artificial constructs as 3D models, that recapitulate crucial aspects of the native cellular microenvironment, has been found mandatory to realize in vitro cell culture which overcome the traditional unnatural 2D paradigm, and, in perspective, valuable substitutes for tissues and organs compromised by trauma or diseases. The most recent challenge of Tissue Engineering techniques, indeed, deals with the design of 3D biomaterials able to mimic the complex structural and biochemical functions of the Extra Cellular Matrix (ECM). Then, these scaffolds have to be populated with the recipient cells providing an informative microenvironment mimicking a physiological niche. The realization of an ECM-like environment is an ambitious goal for the science of biomaterials, considering its complexity in terms of three-dimensional structure of the constituent structural proteins (e.g., collagen); the overall nanofibrous structure with peculiar biomechanical features; and the presence of several adhesive proteins, growth factors, and glycosaminoglycans, each promoting specific cellular activities and for which the local concentration is finely modulated by a mutual ratio of cell–matrix interaction. In coping with this challenge, the international research is gradually developing more and more complex matrices from a structural point of view not neglecting the ability to convey important biochemical stimuli to the cellular component. In recent years, much attention has been paid to formulate matrices characterized by a nanofibrous surface that mimics the ECM. In addition to lithography techniques for nanopatterning and electrospinning, the self-assembly is emerging as bio-inspired technique. It is well known, in fact, that Nature often uses this strategy to get nano- and micro-meter structures built from very small molecules by means of intermolecular forces, primarily consisting in hydrogen bonds and Van der Waals interactions. Self-Assembling Peptides (SAPs) are an example of such molecules: discovered in 1992 by Zhang, these fragments of the protein Zuotin are able to form in solution very stable β-sheet structures [1,2]. The β-sheet layers have a hydrophilic face and a hydrophobic face and can stack each other through ionic interactions and Van der Waals bonds forming hydrogels (a scheme that describes the self-assembly of the peptides is shown in Figure 1). There are many conditions that determine and influence the process among them, the concentration of the peptide in solution, the presence of monovalent positive ions, the pH, the temperature and time. These hydrogels have been used as scaffolds for the growth of different types of cells [3,4,5,6,7] and a particular type of self-assembling peptides is marketed with the name of PuraMatrix^®^. The nanofibrous matrix, obtained by self-assembly, is characterized by interwoven fibers with length ranging from several hundred nanometers to a few microns [8]. The beneficial behavior of the cells in SAP scaffolds is probably due to their nanometric fibrous structure or to their mechanical properties because these peptides do not bear specific motifs to promote cell adhesion or growth. Another interesting feature of the hydrogels of self-assembling peptides is their ability to “capture” large quantities of water: more than 99% of the assembled structure is composed of water, improving transport of oxygen, nutrient and waste, as well as a realistic transport of soluble factors [9]. Moreover, the low stiffness of the material (≃0.1 kPa) allows cells to penetrate into the SAP hydrogels; the survival of compromised cells or the cell proliferation and growth within them have also been demonstrated [10,11]. 

In this study, we report on the design of second-generation peptide hydrogels, formed by a nanofibrous structure able to reproduce even the wealth of biochemical stimuli typical of the ECM. At the state of the art, two main possibilities can be explored: (i) to incorporate the bioactive molecules (carbohydrates, proteins or peptides) into the hydrogels; or (ii) to covalently graft these molecules to the scaffolds (hydrogels). Both strategies have benefits and drawbacks but only the covalent anchoring ensures the expected bio-effect while the release of the adhesive peptide from the matrix, in which it is embedded, determines the opposite, unwanted effect (adhesion inhibition) [12]. Accordingly, we have chosen the covalent anchoring strategy. 

**Figure 2:**
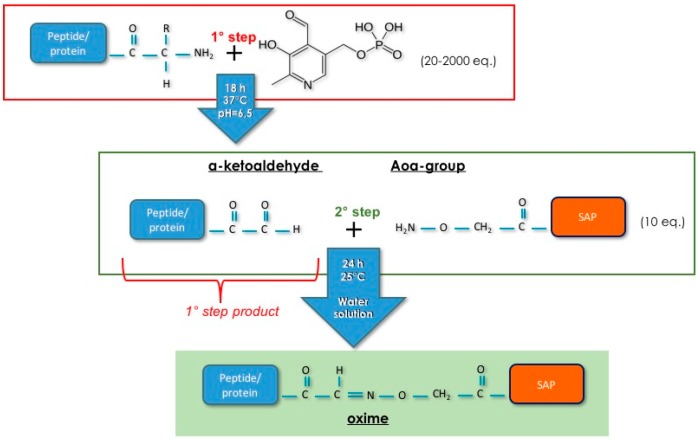
Scheme of chemoselective ligation: Step 1, the bio-transamination with pirydoxal phosphate (PLP) converts the N-terminal Gly in an α-ketoaldehyde; and Step 2, the α-ketoaldehyde reacts with an oxyamino group for producing an oxime.

Developing an effective anchoring strategy implies solving a sequence design problem, i.e., how to get a specific bond between two particular groups in the self-assembling peptide and bioactive molecule. In fact, the side chains of the SAP and those of bioactive peptides/proteins must remain available for the self-assembly and for the interaction with cell receptors, respectively. To meet this need, we used a strategy, known as chemoselective ligation, able to ensure a bio-orthogonal reaction under mild conditions that does not hinder the protein folding. The use of bio-transamination with pirydoxal phosphate (PLP) allows the transformation in the ketone or aldehyde of the first amino acid of peptides/proteins in non-denaturing conditions (Figure 2) [13,14]. The idea that we have formulated is to build highly interwoven nanofibrous architecture in which the cells can submerge themselves and experience a 3D protein-like environment providing biochemical stimuli that promote their adhesion and growth. Despite synthetic porous scaffolds, which offer a minimalist approach to the 3D culture of mammalian cells outside the body, SAP hydrogels act as a permissive template, which have indeed numerous characteristics of the architecture and mechanics of the native cellular microenvironment [15].

The use of conjugates between SAPs and bioactive peptides, integrated into the SAP hydrogel through self-assembling, provides a powerful tool for: (i) decorating the internal part of the hydrogel, and not only its surface; and (ii) testing various concentrations of bioactive molecules to identify biologically active concentrations for a particular cellular system. The bioactive sequences chosen for the study were: (i) a linear peptide of 25 amino acids, henceforth shortly indicated as RGD, containing 4 Glycine-Arginine-Glycine-Aspartic Acid-Serine-Proline (GRGDSP) sequences, that showed adhesive properties for osteoblasts, cardiomyocytes, endothelial cells [12,16,17]; (ii) a peptide that mimics the sequence (351–359) of the human vitronectin that has been shown to promote more and better adhesion of osteoblasts through a mechanism mediated by proteoglycans and only partially by integrins [16,18,19]; and (iii) the protein Insulin-like Growth Factor-1 (IGF-1), a growth factor that dose-dependently stimulated the proliferation of Mesenchymal Stem Cells (MSCs), upregulated the expression of CXCR4, and accelerated migration [20]. The present study proposes the conjugation between a complementary ionic peptide of module II (called EAK) [1] and the above mentioned biologically active molecules. Hydrogels decorated with three concentrations of each conjugate were tested. The hydrogels were seeded with human bone marrow derived MSCs, due to their plasticity and high sensitivity to the physico-chemical properties of the substrates on which they are cultured [21]; cellular adhesion and growth were evaluated after one, four and eight days. The morphology of the functionalized and pristine scaffolds was analyzed by Atomic Force Microscopy, while the effect of conjugate enrichment on the viscoelastic properties of the scaffolds was determined by small amplitude oscillatory shear tests.

## 2. Materials and Methods

### 2.1. Materials

The solid support, Rink Amide MBHA resin, was from Novabiochem (Merck KGaA, Darmstadt, Germany). The Fluorenylmethyloxycarbonyl (Fmoc) protected amino acids were from Novabiochem. The coupling reagents 2-(1H-Benzotriazole-1-yl)-1,1,3,3-tetramethyluronium hexafluorophosphate (HBTU), 1-[Bis(dimethylamino)methylene]-1H-1,2,3-triazolo[4,5-b]pyridinium 3-oxid hexafluorophosphate (HATU), 1-Hydroxybenzotriazole (HOBt) and 3H-[1,2,3]-Triazolo[4,5-b]pyridin-3-ol (HOAt) were from Advanced Biotech (Seveso, Italy). *N*,*N*-diisopropylethylamine (DIEA) and piperidine were from Biosolve (Leenderweg, Valkenswaard, The Netherlands). 2,4,6-Collidine was from Janssen Chimica NV (Beerse, Belgium). Triethoxysilane (TES) was from Sigma-Aldrich (Steinheim, Germany). Solvents such as *N*,*N*-dimethylformamide (DMF), trifluoroacetic acid (TFA), *N*-methyl-2-pyrrolidone (NMP) and dichloromethane (DCM) were from Biosolve. Increlex (10 mg/mL IGF-1 solution) was from Ipsen Pharma (Boulogne-Billancourt, France).

### 2.2. Peptide Synthesis and Conjugate Preparation

#### 2.2.1. EAK

The synthesis of EAK (H-Ala-Glu-Ala-Glu-Ala-Lys-Ala-Lys-Ala-Glu-Ala-Glu-Ala-Lys-Ala-Lys-NH_2_) was carried out on 0.72 mmol/g Rink Amide MBHA resin using Fmoc chemistry by a Syro I synthesizer [22]. The side chain protecting groups were: OtBu, Glu and Boc, Lys. The loading of the first amino acid was carried out with a double coupling. The following four insertions were carried out with single couplings (5 equivalents of Fmoc-amino acid, 5 eq. HBTU, 5 eq. HOBt and 10 eq. DIEA, for 45 min) and the remaining ones with double couplings. At the end of the synthesis the last inserted amino acid was Fmoc-deprotected. Crude peptide was detached from the resin and protecting groups were removed using a 95% TFA, 2.5% TES, 2.5% H_2_O MilliQ (*v*:*v*:*v*). Purification of the crude product was performed through reverse phase high performance liquid chromatography (RP-HPLC). The homogeneity (>99%) of the purified product was obtained by integration of the analytical HPLC peaks, whereas the identity of each product was ascertained by ElectroSpray Ionization-Time Of Flight (ESI-TOF, Mariner System 5220, Applied Biosystem, Perkin-Elmer, Analytical Instruments, Norfolk, CT, USA). Mass data: experimental (exp.) mass = 1614.84 Da; theoretical (theor.) mass = 1614.83 Da. 

#### 2.2.2. Aoa-EAK

The peptide Aoa-EAK (sequence: NH_2_-O-CH_2_-CO-Ala-Glu-Ala-Glu-Ala-Lys-Ala-Lys-Ala-Glu-Ala-Glu-Ala-Lys-Ala-Lys-NH_2_) was synthesized as above reported on Rink Amide MBHA resin (0.7 mmol/g). The last condensation with BisBoc-Aoa-OH, was a double coupling with 5 eq. HATU, 5 eq. HOAt and 10 eq. 2,4,6-collidine. The peptide was deprotected from the resin and side chain protecting groups using the mixture 4.75 mL TFA, 0.125 mL TES, and 0.125 mL H_2_O, for 1.5 h. The identity and homogeneity of crude peptide was ascertained by mass (exp. mass = 1687.92 Da; theor. mass = 1687.87 Da, ESI-TOF) and RP-HPLC analyses. This crude peptide was used for the synthesis of three conjugates with bioactive sequences through chemoselective ligation approach.

#### 2.2.3. G7(GRGDSP)_4_K

The RGD peptide (sequence: H-Gly-7-aminoheptanoic acid-(Gly-Arg-Gly-Asp-Ser-Pro)_4_Lys-NH_2_) was synthesized by standard Fmoc chemistry using Rink Amide MBHA resin (0.7 mmol/g; scale 0.125 mmoles) and a fully automated peptide synthesizer (Syro I, Multisynthec, Witten, Germany). The side chain protection employed were: Arg, Pbf; Asp, OtBu; Ser, tBu; and Lys, Boc. The Fmoc removal was accomplished by two treatments with 40% and 20% piperidine/DMF for 3 min and 12 min, respectively. The coupling reaction was carried out using 5 eq. of Fmoc protected amino acid and HOBt/HBTU/DIPEA (5 eq. HOBt/HBTU and 10 eq. DPEA; 45 min) in DMF. The loading step was carried out with a double coupling, the following four couplings were single and the remaining 22 cycles were double. After the Fmoc deprotection the resin was washed with DCM and dried for 1 h under vacuum. The peptide was cleaved from the solid support with the contemporary side-chain deprotection using the following mixture: 0.125 mL H_2_O MilliQ, 0.125 mL TES, and 4.750 mL TFA (90 min, under magnetic stirring). After cleavage, the resin was filtered, the reaction mixture concentrated and the crude peptide precipitated with cold ethyl ether. Forty milligrams of crude peptide dissolved in 20 mL of MilliQ water were loaded on Jupiter C_18_ column (5 µm, 300 Å, 10 × 250 mm, Phenomenex, Torrance, CA, USA) and separated in the following conditions: eluent A, 0.05% TFA in MilliQ water; eluent B, 0.05% TFA in CH_3_CN; gradient, 0%–6% B in 2 min, then 6%–14% B in 32 min; flow rate, 4 mL/min; detection at 214 nm.

#### 2.2.4. α-Ketoaldehyde-7-Aminoheptanoicacid-(GRGDSP)_4_K

The conversion of G7(GRGDSP)_4_K in α-ketoaldehyde-7-aminoheptanoic acid-(GRGDSP)_4_K (sequence: H-CO-CO-NH-(CH_2_)_6_-CO-(Gly-Arg-Gly-Asp-Ser-Pro)_4_Lys-NH_2_) was obtained by addition of 23.63 mg of G7(GRGDSP)_4_K (9.061 × 10^−6^ moles) to 18.12 mL of 10 mM pyridoxal phosphate (PLP) in 25 mM sodium phosphate buffer pH 6.5 (181.23 × 10^−6^ moles PLP; G7(GRGDSP)_4_K:PLP = 1:20) for 18 h at 37 °C. The α-ketoaldehyde-peptide was isolated by RP-HPLC and characterized by analytical RP-HPLC (conditions: Symmetry Shield C_8_ column (5 µm, 100 Å, 4.6 × 250 mm, Waters), eluent A: 0.05% TFA in H_2_O; eluent B: 0.05% TFA in CH_3_CN; gradient: 0%–25% di B in 25 min, flow rate: 1 mL/min; detector: 214 nm. *t*_R_ = 16.7 min) and ESI mass spectrometry (theor. mass, 2606.27 Da; exp. mass, 2606.27 Da, ESI-TOF).

#### 2.2.5. SAP-RGD

The chemoselective ligation between α-ketoaldehyde-7-aminoheptanoic acid-(GRGDSP)_4_K and Aoa-EAK produced the conjugate named SAP-RGD. Breafly, 25.894 × 10^−6^ moles of Aoa-EAK was added to 2.589 × 10^−6^ moles of α-ketoaldehyde-7-aminoheptanoic acid-(GRGDSP)_4_K (ratio: 10:1) in 25.89 mL of H_2_O MilliQ for 24 h at room temperature. The product was isolated using RP-HPLC in the following conditions: Jupiter C_18_ column (5 µm, 300 Å, 10 × 250 mm, Phenomenex); eluent A: 0.05% TFA in H_2_O; eluent B: 0.05% TFA in CH_3_CN; gradient: 0%–5% di B in 2 min, 5%–35% B in 60 min, then at 80% B for 10 min; flow rate: 4 mL/min; detector: 214 nm. The identity of the product was confirmed by MALDI mass analysis (theor. mass 4277.61 Da; exp. mass: 4278.54 Da). In analytical chromatography (conditions: column: Jupiter C_18_; flow rate: 1.0 mL/min; eluent A, 0.05% TFA in H_2_O MilliQ; eluent B, 0.05% TFA in CH_3_CN; gradient 0%–35% B in 35 min, detector at 214 nm) the conjugate SAP-RGD showed a *t*_R_ = 21.7 min.

#### 2.2.6. G7HVP

The peptide G7HVP (sequence: H-Gly-7-aminoheptanoic acid-Phe-Arg-His-Arg-Asn-Arg-Lys-Gly-Lys-NH_2_) was synthesized by standard Fmoc chemistry using Rink Amide MBHA resin (0.7 mmol/g; scale 0.125 mmoles) and a fully automated peptide synthesizer (Syro I, Multisynthec, Witten, Germany). The side chain protection employed were: Arg, Pbf; Asn and His, Trt; Tyr, tBu; and Lys, Boc. The Fmoc removal was accomplished by two treatments with 40% and 20% piperidine/DMF for 3 min and 12 min, respectively. The coupling reaction was carried out using 5-fold excess of Fmoc protected amino acid and HOBt/HBTU/DIPEA (5 eq. HOBt/HBTU and 10 eq. DPEA; 45 min) in DMF. All of the couplings were double. After the Fmoc deprotection the resin was washed with DCM and dried for 1 h under vacuum. The peptide was cleaved from the solid support with contemporary side-chain deprotection using the following mixture: 0.125 mL H_2_O MilliQ, 0.125 mL TES, and 4.750 mL TFA (90 min, under magnetic stirring). After cleavage, the resin was filtered, the reaction mixture concentrated and the crude peptide precipitated with cold ethyl ether. Thirty milligrams of crude peptide dissolved in 15 mL of MilliQ water were loaded on Jupiter C_18_ (5 µm, 300 Å, 10 × 250 mm, Phenomenex) and separated in the following conditions: eluent A, 0.05% TFA in MilliQ water; eluent B, 0.05% TFA in CH_3_CN; gradient, 0%–10% B in 2 min, then 10%–30% B in 40 min; flow rate, 4 mL/min; detection at 214 nm.

#### 2.2.7. α-Ketoaldehyde-7-Aminoheptanoic Acid-HVP

The conversion of G7HVP in α-ketoaldehyde-7-aminoheptanoic acid-HVP (sequence: H-CO-CO-NH-(CH_2_)_6_-CO-(Phe-Arg-His-Arg-Asn-Arg-Lys-Gly-Lys-NH_2_) was obtained by addition of 9.96 mg of G7HVP (7.034 × 10^−6^ moles) to 14.06 mL of 10 mM pyridoxal phosphate (PLP) in 25 mM sodium phosphate buffer pH 6.5 (1.4068 × 10^−4^ moles PLP; G7HVP:PLP = 1:20) for 18 h at 37 °C. The chromatogram of purified peptide was obtained in the following conditions: column, Jupiter C_18_ (5 µm, 300 Å, 4.6 × 250 mm, Phenomenex); injection volume, 20 µL of 1 mg/mL peptide solution; flow rate, 1 mL/min; eluent A, 0.05% TFA in water; eluent B, 0.05% TFA in CH_3_CN; gradient, 10%–30% B in 20 min, detection at 214 nm. The retention time results 12.7 min and the purity grade, 82.6%. Exp. mass: 1414.7 Da, theor. mass: 1415.6 Da (ESI-TOF).

#### 2.2.8. SAP-HVP

The chemoselective ligation between α-ketoaldehyde-7-aminoheptanoic acid-HVP and Aoa-EAK produced the conjugate named EAK-HVP. An amount of 14.81 × 10^−6^ moles of Aoa-EAK was added to 1.481 × 10^−6^ moles of α-ketoaldehyde-7-aminoheptanoic acid-HVP (ratio, 10:1) in 14.81 mL of H_2_O MilliQ for 24 h at room temperature. The product was isolated using RP-HPLC in the following conditions: Jupiter C_18_ column (5 µm, 300 Å, 10 × 250 mm, Phenomenex); eluent A: 0.05% TFA in H_2_O; eluent B: 0.05% TFA in CH_3_CN; gradient: 0%–5% di B in 2 min, 5%–35% B in 60 min, then at 80% B for 10 min; flow rate: 4 mL/min; detector: 214 nm. The identity of the product was confirmed by ESI mass analysis (theor. mass 3085.54 Da; exp. mass: 3085.64 Da). In analytical chromatography (conditions: column: Jupiter C_18_; flow rate: 1 mL/min; eluent A, 0.05% TFA in H_2_O MilliQ; eluent B, 0.05% TFA in CH_3_CN; gradient 10%–30% B in 20 min, detector at 214 nm), the conjugate SAP-HVP showed a *t*_R_ = 14.8 min.

#### 2.2.9. α-Ketoaldehyde-IGF-1

The conversion of IGF-1 in α-ketoaldehyde-IGF-1 was obtained by addition of 10 mg of IGF-1 (1.307 × 10^−6^ moles) to 26.3 mL of 100 mM pyridoxal phosphate (PLP) in 25 mM sodium phosphate buffer pH 6.5 (2.615 × 10^−3^ moles PLP; IGF-1:PLP = 1:2000) for 3 h at 37 °C and 1 h at room temperature. The chromatogram of purified peptide was obtained in the following conditions: column, Vydac C_18_ Proteo & Peptide (5 µm, 300 Å, 4.6 × 250 mm, Grace); injection volume, 20 µL of 1 mg/mL peptide solution; flow rate, 1 mL/min; eluent A, 0.05% TFA in water; eluent B, 0.05% TFA in CH_3_CN; gradient, 20%–40% B in 40 min, detection at 214 nm. The retention time results 29.4 min and the purity grade is over 80%. Exp. mass: 7648 Da, theor. mass: 7648 Da (ESI-TOF). 

#### 2.2.10. SAP-IGF-1

The chemoselective ligation between α-ketoaldehyde-IGF-1 and Aoa-EAK produced the conjugate named SAP-IGF-1. An amount of 5.62 × 10^−6^ moles of Aoa-EAK was added to 0.562 × 10^−6^ moles of α-ketoaldehyde-IGF-1 (ratio, 10:1) in 5.62 mL of H_2_O MilliQ for 24 h at room temperature. The product was isolated using RP-HPLC in the following conditions: Jupiter C_18_ column (5 µm, 300 Å, 10 × 250 mm, Phenomenex); eluent A: 0.05% TFA in H_2_O; eluent B: 0.05% TFA in CH_3_CN; gradient: 0%–10% di B in 2 min, 10%–50% B in 80 min, then at 80% B for 10 min; flow rate: 4 mL/min; detector: 214 nm. The identity of the product was confirmed by ESI mass analysis (theor. mass 9318.87 Da; exp. mass: 9319.9 Da). In analytical chromatography (conditions: column: Jupiter C_18_; flow rate: 1 mL/min; eluent A, 0.05% TFA in H_2_O MilliQ; eluent B, 0.05% TFA in CH_3_CN; gradient 10%–50% B in 40 min, detector at 214 nm) the conjugate SAP-IGF-1 showed a *t*_R_ = 24.3 min.

### 2.3. Decoration of SAP Scaffolds

Hydrogel scaffolds were prepared dissolving 10.03 mg of SAP (EAK) into 10.03 mL of deionized water (1% *w*/*v*; 6.19 mM) and sonicated for 30 min, and then 20 µL of this solution was placed into each well of cell culture plate. The final concentration of SAP (0.5% *w*/*v*) was obtained adding 20 µL of DMEM to all samples. SAP-RGD, SAP-HVP and SAP-IGF-1 conjugates were used to enrich SAP scaffolds; three different concentrations were tested for each conjugate: 4 × 10^−5^ M, 4 × 10^−6^ M, 4 × 10^−7^ M. 

**Figure 3:**
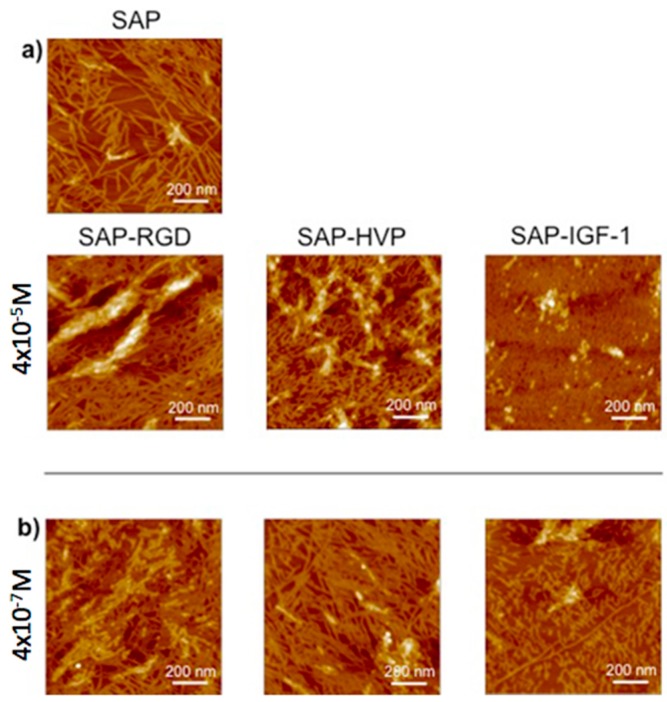
Atomic Force Microscopy (AFM) images of: (**a**) Self-assembling peptide hydrogel pristine (SAP) or enriched with conjugates between SAPs and adhesive peptides (called SAP-RGD and SAP-HVP) or decorated with a conjugate between SAP and Insulin-like Growth factor-1 (called SAP-IGF-1) at 4 × 10^−5^ M; and (**b**) SAP-RGD, SAP-HVP and SAP-IGF-1 at 4 × 10^−7^ M on mica surface.

The scaffold marked with rodhamine (Figure 3) was obtained by using 1% enrichment of purified rhodamine-SAP conjugate. A lower concentration of SAP (0.15% *w*/*v*) was used for AFM samples: the analysis of fiber features is almost impossible at 0.5% (*w*/*v*) for the overlay of several fiber sheets.

### 2.4. Atomic Force Microscopy (AFM)

Atomic Force Microscopy (AFM) observations were carried out with the “J scanner” in tapping mode by using a Nanoscope IIIA-MultiMode AFM (Digital Instruments, Santa Barbara, CA, USA) under room conditions. The force was maintained at the lowest possible value by a continuous adjusting of the set point during the imaging phase. Images were recorded using 0.5–2 Ω·cm phosphorous (n) doped silicon tips mounted on cantilevers with a nominal force constant of 40 N/m, a resonance frequency of 300 kHz and a tip curvature radius of 10 nm. 

### 2.5. Rheological Analysis: Small Amplitude Oscillatory Shear Tests

The viscoelastic properties of SAP and SAP-IGF-1 were evaluated at 37 °C using a rheometer (Gemini, Bohlin Instruments) with parallel-plate geometry. In particular, to avoid slippage, serrated parallel plates (15 mm in diameter) were used. Strain sweep tests were first performed to determine the linear viscoelastic region, and successively small amplitude oscillatory shear tests were carried out. The frequency was varied from 0.01 to 2 Hz.

In the investigated range of frequency, the storage or elastic modulus (*G*′) and the loss or viscous modulus (*G*″) were evaluated as follows:

(1) $$G^{\prime }=\frac{\tau_{0}}{\gamma_{0}}\cos\delta ,$$

(2) $$G^{\prime \prime }=\frac{\tau_{0}}{\gamma_{0}}\sin\delta ,$$

where τ_0_ and γ_0_ are the stress and the strain amplitudes, respectively, and δ is the phase shift between the input and the output signals.

The results were analyzed using ANOVA followed by Bonferroni post-hoc tests and statistical differences were set at *p* < 0.05.

### 2.6. Biological Assays

#### 2.6.1. Cell Culture

Bone marrow-derived mesenchymal stem cells commercially available (ATCC-PCS-500-012, LGC Standards) have been used for biological tests. Cells were expanded in Coon’s modified Ham’s F12 enriched with 10% Fetal Bovine Serum (FBS), 1% l-glutamine and 1% penicillin/streptomycin (all from Sigma-Aldrich), and cultured in incubator at 37 °C with a controlled atmosphere of 5% CO_2_ to allow gas exchange. The medium was changed twice a week. When the required confluence was reached, cells were detached with 0.05% Trypsin (Sigma-Aldrich) and counted. Cells were then suspended in order to obtain a density of 2 million cells/mL. Each gel was generated by adding 20 µL of cell suspension to 20 µL of peptide (40,000 cells per gel). Gelation was allowed to take place at 37 °C for about 10 min; extra culture medium (150 µL per gel) was then added after 1 h. MSCs were cultured on plastic dish as 2D control. All experiments were performed in triplicate.

#### 2.6.2. Biological Validation

Cell proliferation was evaluated after one, four or eight days by using a cell viability reagent (Presto Blue test, Invitrogen, Carlsbad, CA, USA). This assay is based on the ability of the reagent, a resazurin-based solution, to measure the reducing power of living cells as a measure of their proliferation. When added to the cells, the reagent is modified by the reducing environment of the viable cells and turns from blue to red. The assay was added to the culture medium at a concentration of 10% *v*/*v* to each well. Cells-sample constructs were then incubated for 10 min at 37 °C in the dark. The supernatant was removed and its absorbance was quantified by spectrophotometry at 570 and 600 nm. The levels of cell proliferation were expressed as percentage with respect of the control.

Cellular adhesion and morphology into the samples were verified one week after cell seeding by fluorescence optical microscopy analysis (Nikon H550L optical microscope), while cell spatial distribution through the entire 3D gel was investigated by confocal optical microscopy (Leica TCS SP5 AOBS confocal microscope). For all typologies, samples were fixed with 4% paraformaldehyde for 1 h and treated with 0.1% Triton X-100 to permeabilize cell membrane. Nuclei were stained with 4,6-Diamidino-2-phenylindole (DAPI) dye (1 μg/mL), actin filaments were stained with Alexa Fluor 488 phalloidin (100 μM), all by Sigma-Aldrich. Alexa Fluor 488 was excited with the 488 nm line of the Arglaser and its fluorescence was collected in a spectral window of 500 to 530 nm. For DAPI stain acquisition, 720 nm excitation wavelength and 450–500 nm spectral window emission were used.

To value cell distribution in 3D, we collected z-stacks of 30 µm (maximum thickness of the gels) after one day from seeding. Each stack was composed of 30 images at 1 μm z-spacing.

Several features characterizing cell morphology were identified and measured both in 3D and in 2D by using the image analysis software ImageJ (NIH). At least 20 cells were considered for each hydrogel; images were converted to 8-bit images and an automatic threshold was applied to discriminate cells (black) from free space (white). The following parameters were then considered and measured for each cell: area of the cell, perimeter of the cell, major axis and minor axis of the best fitting ellipse.

#### 2.6.3. Statistical Analysis

**Figure 4:**
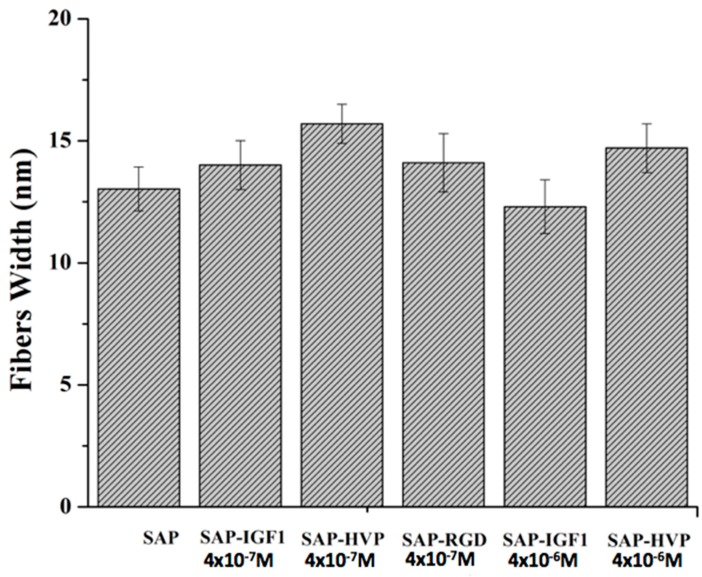
Fibers width of Self-Assembling Peptide (SAP) hydrogels and decorated SAP hydrogels at different concentrations.

**Figure 5:**
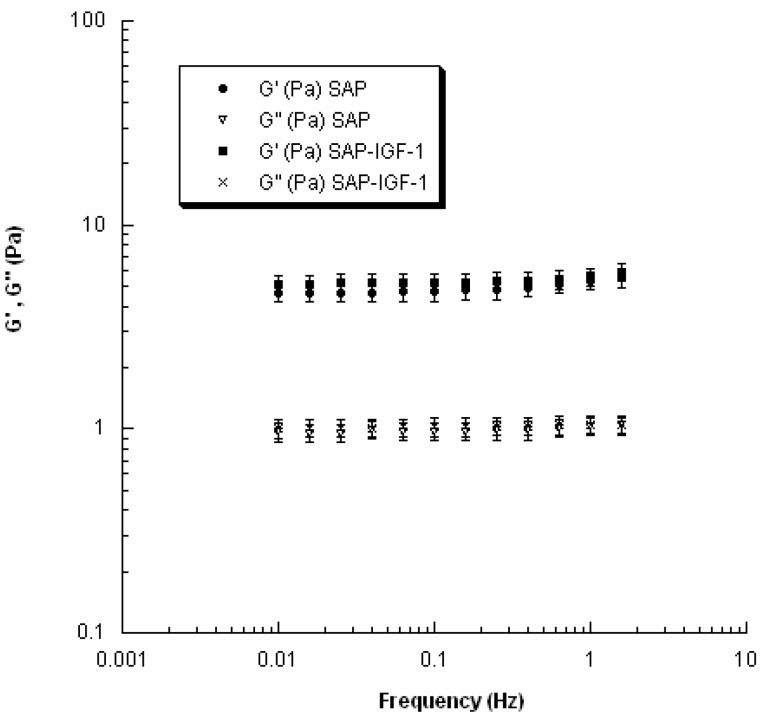
Storage modulus (*G*′) and loss modulus (*G*″) as function of frequency for SAP and SAP-IGF-1 at concentration of 4 × 10^−5^ M. Data are graphically reported as mean value, and error bars represent the standard deviation. No statistically significant differences were found between SAP and SAP-IGF-1 in the investigated range of frequency.

Statistical analysis was performed on cell proliferation results (Figure 4 and Figure 5), to evaluate the differences between: (i) functionalized-hydrogels and SAP control; (ii) hydrogels functionalized with the same conjugate, but with different concentrations; and (iii) hydrogels functionalized with different conjugates, at the same concentration.

All numerical data are presented as mean and standard deviation. Statistical evaluation was performed using non-parametric Mann–Whitney test to determine significant differences between groups. The significance level was set at *p* < 0.05.

## 3. Results

### 3.1. Synthesis of Conjugates

The first step of chemoselective ligation approach (Figure 2) was the bio-transamination reaction. In this paper, we have converted into alpha-ketoaldehyde two adhesive peptides (G7HVP and G7(GRGDSP)_4_K) in which the bioactive motif was condensed with the dipeptide G7 (H-Gly-7-amino-heptanoic acid). In a previous study [23], we demonstrated that the bio-transamination and the following chemoselective ligation via oxime increased their yields by the addition of this spacer at N-terminus of the peptides. The positive effect of G7 dipeptide was demonstrated for (GRGDSP)_4_K sequence in consideration of the same N-terminal residue of this peptide and the G7(GRGDSP)_4_K analog. It is well known that the identity of N-terminal residue is important for transamination outcome and in particular that Ala, Gly, Asp, Gln and Asn residues led to very high conversions [13,14].

### 3.2. Hydrogel Morphology at the Nanoscale

Figure 3a shows that the process of self-assembly is strikingly different for SAP and the three different conjugates SAP-HVP, SAP-RGD and SAP-IGF-1 at the different concentration employed in the work (i.e., 4 × 10^−5^ M, 4 × 10^−6^ M and 4 × 10^−7^ M). In particular, the main effect of conjugates addition is the inhibition of long fibers, which are in fact observed for SAP, but not for the decorated hydrogels, at all of the concentrations. In detail, the morphology of the hydrogel structure decorated with the highest conjugate concentration, 4 × 10^−5^ M, is deeply different for the three conjugates. In fact, the SAP-RGD hydrogel is formed by a dense multilayer of interwoven relatively long fibers, similar to pure SAP, the SAP-HVP hydrogel consists in a sparse dispersion of short nanofibrils, and the SAP-IGF-1 hydrogel yields a compact and apparently unstructured layer of peptides, showing very dense interwoven tissue of fibrils and tiny nanopores.

The concentration basically affects the degree of coverage of the substrate, yielding dense structures at the highest concentrations, and sparse distributions of fibers or fibrils at the lowest ones (4 × 10^−7^ M). In all the cases, evidences of a three-dimensional nanofibrous structure can be seen, with a two-level system consisting in an underlayer with quite compact networks, narrow and high cross-linking density fibrils, and an overlayer consisting of less dense networks, with larger fibrils and larger meshes.

In order to understand the structure of the SAP and SAP-conjugate moieties, the images of the hydrogel phases at the lowest concentration have been obtained (see Figure 3b). All the hydrogels obtained at very low concentration show very peculiar common features: the fibers or fibrils are very similar in lateral width for all the SAP sequences. Figure 4 reports the histogram obtained by taking the dimensions of several structures per image. The width for all the structures ranges between 12 and 16 nm, depending on the nature of the conjugating groups. These values are very close to the 13.0 ± 2.0 nm measured here for the characteristic long fibers of pure SAP formed at concentration of 4 × 10^−5^ and 4 × 10^−6^ M. As the “nominal” length of a fully extended SAP sequence is 5.6 nm, the experimentally measured value can be interpreted in terms of a side-by-side aggregation mechanism of two ~ 6.0 ± 1.0 nm long SAP sequences, giving the width of ~12.0 ± 1.0 nm, and by the further pile-up of this elementary binary building block in more or less long sequences. The assembling process is clearly driven by the alternating self-complementary nature of the basic SAP segments, while the pendant conjugated peptides basically hinder the longitudinal growth of the fibers. 

### 3.3. Rheological Analysis: Small Amplitude Oscillatory Shear Tests

Results from small amplitude shear tests are reported in Figure 5. 

In the investigated range of frequency, *G*′ values were always higher than *G*″ ones for both SAP and SAP-IGF-1 (4 × 10^−5^ M). Specifically, with regard to SAP, G′ ranged from 4.7 ± 0.4 Pa to 5.7 ± 0.5 Pa by increasing the frequency from 0.01 to 2 Hz, while SAP-IGF-1 provided *G*′ values spanning from 5.0 ± 0.4 Pa to 5.9 ± 0.5 Pa.

However, the presence of IGF-1 did not alter the viscoelastic properties of the SAP (EAK) as no statistically significant differences were found between SAP and SAP-IGF-1 in the investigated range of frequency.

### 3.4. Cell Distribution within the 3D Gels

**Figure 6:**
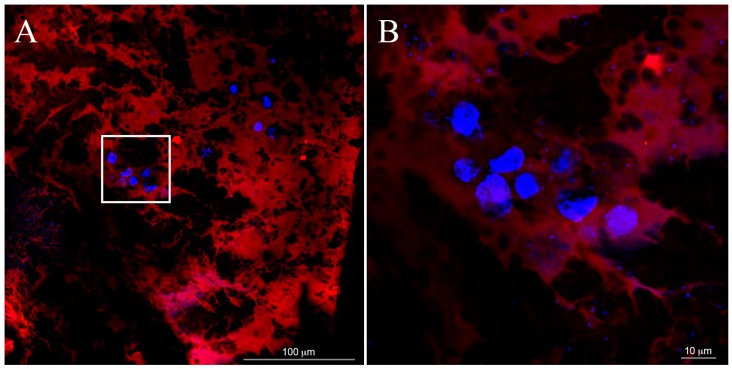

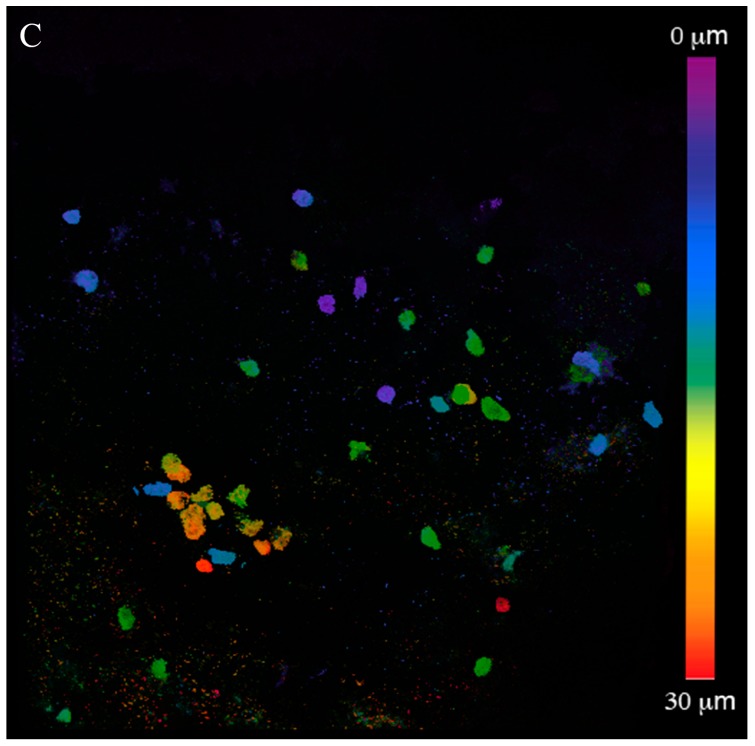
Mesenchymal Stem Cell (MSC) nuclei (blue, 4′-6-DiAmino-2-PhenylIndole-DAPI) adhering to the SAP fibers of the hydrogel (red, Rodhamin) one day after seeding (**A**,**B**) (zoom); and (**C**) the nuclei distribution through the entire thickness of the gel (projection). Nuclei with different colors are placed at different levels (from 0 µm (purple nuclei) to 30 µm (red nuclei)).

Cellular distribution through the hydrogels was investigated by confocal microscopy on Day 1. As shown in Figure 6A,B (zoom), several cells were found adhering to the filament structure of the material (a single layer is shown as representative image). A projection of images captured through the entire thickness of the gel is shown in Figure 6C. Nuclei characterized by different colors corresponding to different levels are clearly observable, meaning that cells successfully colonized the overall hydrogel.

### 3.5. Cell Proliferation and Morphology

**Figure 7:**
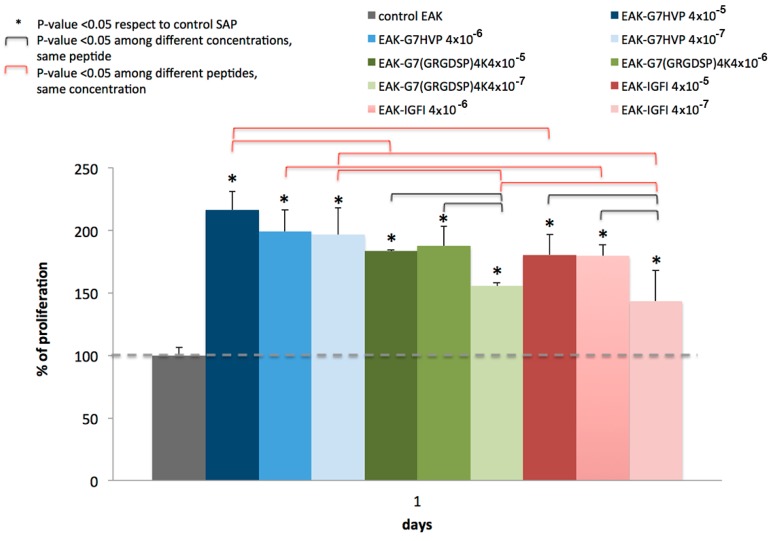
Percentage of cellular proliferation after one day, as indication of Mesenchymal Stem Cell (MSC) adhesion, normalized to the Self-Assembling peptide (SAP) control.

By loading an equal number of MSCs, after just one day, we observed an enhanced, statistically significant, adhesion and viability of cells in all the functionalized hydrogels if compared to the control (non-functionalized peptides based hydrogels); this initial cellular adhesion was also sensible to the concentration of the peptides, as reported in Figure 7. Differences in cell proliferation among different gel types were also observable: in detail, hydrogels enriched with SAP-HVP showed the best results for every concentration here considered (statistically different from SAP-RGD and SAP-IGF-1 at 4 × 10^−5^ M, statistically different from SAP-IGF-1 at 4 × 10^−6^ M, statistically different from SAP-RGD and SAP-IGF-1 at 4 × 10^−7^ M).

**Figure 8:**
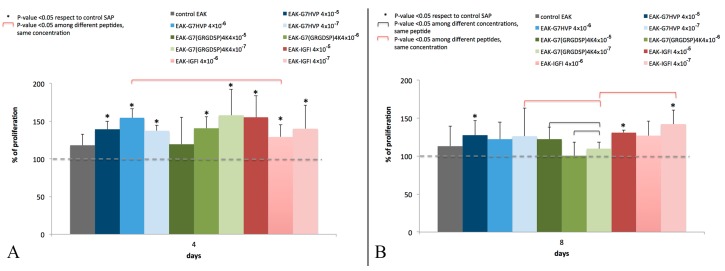
Percentage of cellular proliferation after four days (**A**); and eight days (**B**), as indication of Mesenchymal Stem Cell (MSC) adhesion, normalized to the Self-Assembling Peptide (SAP) control.

After four days, all decorated gels still displayed their ability to support the cellular proliferation, that was statistically higher than the control (Figure 8A); after eight days, this trend appeared toned down, even though a general enhancement in cell proliferation in functionalized gels with respect to the control was maintained, especially in hydrogels functionalized with SAP-IGF-1 (Figure 8B).

**Figure 9:**
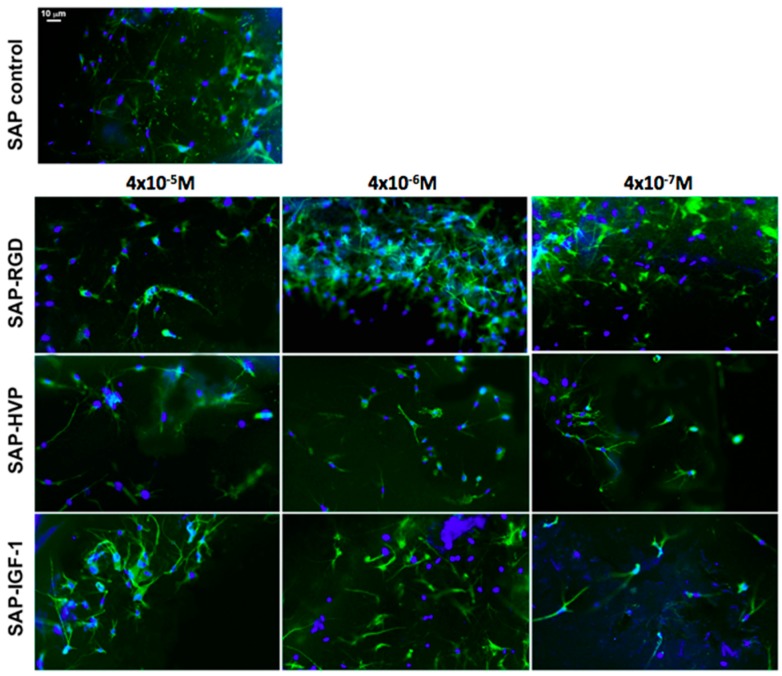
Fluorescence microscopy images of Mesenchymal Stem Cells (MSCs) within plain hydrogel (**top**) or hydrogel enriched with the conjugate called SAP-RGD (**left**); hydrogel enriched with the conjugate called SAP-HVP (**middle**); and hydrogel enriched with the conjugate called SAP-IGF-1 (**right**). The different conjugate concentration is reported on the left side of the picture. Cells show highly elongated shape in all hydrogel types (blue: 4′-6-DiAmino-2-PhenylIndole-DAPI-nuclei, green: Phalloidin-actin filaments). Scale bar is the same as the control for all images.

The fluorescence microscopy analysis showed that cells maintained a highly elongated shape and a pronounced capability of arranging themselves within the hydrogels (Figure 9), probably due to the “soft” mechanical and excellent chemical properties of the bulk hydrogel. In that context, no particular differences were noticed in cell morphology among different gel types. 

**Figure 10:**
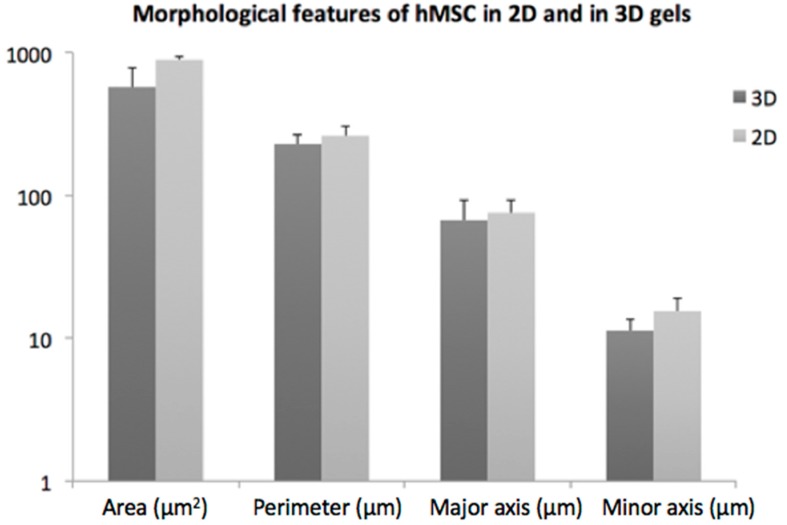
Histogram of Mesenchymal Stem Cells (MSCs) cultured either within 3D Self-Assembling Peptide (SAP) hydrogels or in 2D. No significant differences were observed by quantifying some morphological features of cells.

This elongated shape was quantified by measuring some cell morphological features both in 3D (hydrogel) and in 2D (plastic optimized for cell adhesion) as control condition. Results show that MSC embedded in these SAP hydrogels display values of area, perimeter and circularity (i.e., major and minor axis) very close to those measured for MSC expanded in established cell culture conditions (i.e., 2D) (Figure 10); on the other hand, the cells, only when completely immersed in the hydrogel, can stretch in a three-dimensional “physiological” environment.

## 4. Discussion

The structure of the nanofibers at the nanometer level was evaluated by AFM. Each enrichment with the conjugates amends the hydrogel morphology, which is influenced by both the nature of the conjugate and its concentration. The most important observation concerns the width of the fibers, which is ranging in a rather narrow range between 12 and 16 nm; otherwise, the length of the fibers seems very affected by the addition of the conjugates. Unfortunately, it was not possible to study the morphological characteristics of the hydrogels using the same concentration of the biological assays and rheology because, at the concentration 0.5% *w*/*v*, an effect of saturation is observed, and consequently the fibers are not visible. Certainly the growing concentration further modifies the scenario: the density of fibril networks of EAK16-II on mica increases sharply up to 0.2 mg/mL concentration and more slowly for higher concentration, whereas the fibril width seems to increase moderately with concentration [24]. Consequently, a strictly correlation between morphological characterization at 0.15% *w*/*v* SAP concentration and biological data obtained at 0.5% *w*/*v* SAP concentration is not possible. In overall, the AFM data are in agreement with recent SEM findings, showing that additional active motifs appended to SAP backbone do not affect nanofiber structures, which in fact remains quite similar to the one observed for pure SAP [5,8]. On the other hand, the presence of our conjugates within the scaffold does not alter the viscoelastic properties of the hydrogels as evident from the values of G′ and G″ obtained for SAP-IGF-1-enriched hydrogel at the maximum concentration tested (4 × 10^−5^ M). The result is in agreement with data reported in the work of Jung and coworkers [25]. SAP-IGF-1 was chosen as a model for rheological characterization because it is the conjugate with the greatest molecular weight among the conjugates synthesized. 

MSCs are immunomodulatory, multipotent and fast proliferating and these unique capabilities mean they can be used for a wide range of treatments including bone, cartilage or myocardium regeneration [21]. In cell therapy, the fate of transplanted cells is the critical factor: few of the stem cells are able to survive [26] and their behavior is deeply affected by the microenvironment where they are forced to be cultured [27]. The self-assembling peptides (SAP) have been used as scaffolds for cell delivery: in the article of Cui et al., MSCs mixed with the SAP RAD 16-II resulted more effective to promote myocardial regeneration in a rat model of myocardial infarction [28]. In addition, SAPs were also used to promote functional protein delivery [29]. As 3D models for investigating in vitro cell biology and physiology, alternative to 2D cell cultures, SAP hydrogels have displayed offering to the cells a permissive template for their migration, proliferation and differentiation. Moreover, they can incorporate active peptide sequences from desired proteins, allowing the controlled placement of specific binding domains [30].

Among the factors that stimulate in vitro MSCs migration and proliferation without influencing their differentiation, there is the insulin-like growth factor 1 (IGF-1) [20]. In the treatment of myocardial infarction, SAP carrying IGF-1 (SAP and IGF-1 were biotynilated and the streptavidin protein was used to create a molecular sandwich) was adopted to make cytokine play a long-term therapy role [31].

The conjugates here reported present some peculiarities with respect to similar molecules described in literature: (i) a spacer (7-amino-heptanoic acid) between the bio-active peptide and the SAP instead of the Gly spacer, proposed by Taraballi et al. [32] able to improve both the stability of the scaffold and the adhesion/proliferation of the cells; (ii) a covalent selective bond connecting the SAP with the bio-active molecule instead of sandwich approaches; (iii) SAP conjugation with an adhesive peptide carrying 4 Glycine-Arginine-Glycine-Aspartic Acid-Serine-Proline (GRGDSP) motifs (GRGDSP is the adhesive motif mapped on fibronectin) instead of the use of peptide with two Arginine-Glycine-Aspartic Acid (RGD) sequences [33]; and (iv) the first proposed SAP conjugate with an adhesive peptide mapped on h-vitronectin. Here, we have demonstrated that hydrogels decorated with the three different conjugates (SAP-HVP, SAP-RGD and SAP-IGF-1) significantly promote MSC adhesion with respect to SAP hydrogels. In particular, the hydrogel decorated with HVP conjugate gives the best results at all the concentrations tested: HVP enriched hydrogels resulted significantly more efficient in the promotion of MSC adhesion in the early times of interaction (Day 1) with respect to IGF-1 enriched samples, in agreement with HVP nature of adhesion promoter. Moreover, we have shown that the effect on MSC adhesion seems increase with every conjugate concentration for all proposed conjugate after one day of in vitro culture. These differences appear toned down after four and eight days; however, a general enhancement in cell proliferation in functionalized gels with respect to the control is maintained, especially in hydrogels functionalized with IGF-1 and the observation is in agreement with the role of growth factor of IGF-1. In literature, some studies demonstrated that IGF-1 system may stimulate osteoblast-like cells proliferation [34] and differentiation [35]. It has also shown IGF-1 inducing intracellular signalling activation in MIO-M1 cells and their migration [36].

We have shown the HVP peptides significantly enhance the initial cellular adhesion of human MSC, better than the RGD sequence. RGD is the principal ligand for the integrin family of adhesion receptors on fibronectin and vitronectin that include the α5β1 and αvβ3 [37,38]. Integrin receptors also recognize other protein domains, such as the heparin-binding (HB) sites, that contribute to the cell adhesion and the formation of the cytoskeleton.

In our hydrogels, cytoskeletal elongation was similar to that typically observed in MSC cultured in 2D on plastic optimized for cell adhesion: the penetration of the cells in hydrogels, however, ensures an elongation in three dimensions rather than in two dimensions. The cytoskeletal elongation detected in our hydrogels is markedly more pronounced with respect to what has been observed in traditional gel cultures, where cells usually show a packed, round-like shape [39,40,41]. This fact is symptomatic of an improved cellular adhesion given by the functionalization of the 3D hydrogels, in addition to their stiffness [9].

This polarized cytoskeleton orientation suggests a biomimetic cellular interaction with the overall neighboring matrix, while in 2D cultures only a segment of the cellular membrane can interact with the ECM and the rest of the cell is exposed to the bulk culture media [9]. Cellular morphology has been shown influencing other cellular processes such as proliferation and gene expression [42]. Thus, a proper design of 3D SAP hydrogels that recapitulate critical mechanical and biochemical cues offers to the cells, as shown here, a microenvironment facilitating cellular migration and tissue organization. Moreover, it is worth noting that the presence of the growth factor IGF-1 did not alter the viscoelastic properties of the SAP as demonstrated by rheological analysis.

In addition, the morphological features of MSCs embedded within gels suggest a mechanobiology of gels permissive to mass transport and cytoskeleton elongation.

This important feature opens the possibility of using these peptides-based gels as powerful 3D cell culture models, more realistic than traditional 2D cultures which fail to reproduce some crucial aspects of cells physiology and morphogenesis in vitro, such as cell–matrix interactions and migration cell capability [43]. The composition and presentation of specific ligands on a substrate, in addition to its stiffness, have been also shown to influence MSC differentiation, modulating the expression of markers associated with neurogenesis, myogenesis or osteogenesis [44]. The study of cells within a 3D environment will thus lead to advances in areas such as cell therapy, tissue engineering and fundamental cell biology. Future studies will address the combination of multiple bioactive motifs into 3D SAP hydrogel architecture and to probe conjugate concentrations greater than 4 × 10^−5^ M, for the fulfillment of other requirements in biology. In particular, cellular differentiation and metabolomics studies could be taken into account, especially when specific ligands triggering selective commitments will be adopted.
